# Translational aspects in targeting the stromal tumour microenvironment: From bench to bedside

**DOI:** 10.1016/j.nhtm.2016.03.001

**Published:** 2016-01

**Authors:** R. Bhome, H.A. Al Saihati, R.W. Goh, M.D. Bullock, J.N. Primrose, G.J. Thomas, A.E. Sayan, A.H. Mirnezami

**Affiliations:** a Cancer Sciences, Faculty of Medicine, University of Southampton, Somers Cancer Research Building, Southampton General Hospital, Tremona Road, Southampton SO16 6YD, UK; b University Surgery, South Academic Block, Southampton General Hospital, Tremona Road, Southampton SO16 6YD, UK; c School of Medicine, University of Southampton, University Road, Southampton SO17 1BJ, UK

**Keywords:** TME, tumour microenvironment, MSC, mesenchymal stem cell, NK, natural killer, APC, antigen presenting cell, EC, endothelial cell, ECM, extracellular matrix, CAF, cancer-associated fibroblast, EMT, epithelial–mesenchymal transition, αSMA, alpha smooth muscle actin, LOX, lysyl oxidase, LOX-L, lysyl oxidase-like protein, BAPN, beta-aminopropionitrile, FGF, fibroblast growth factor, VEGF, vascular endothelial growth factor, Th, helper T cell, Treg, regulatory T cell, TIL, tumour infiltrating lymphocyte, IFN, interferon, CTLA-4, cytotoxic T lymphocyte-associated protein-4, PD-1, programmed cell death protein-1, PD-L1, PD-1 ligand, NSCLC, non-small cell lung cancer, TAM, tumour associated macrophage, CSFR-1, colony stimulating factor receptor-1, PBMC, peripheral blood mononuclear cell, PDGF-β, platelet-derived growth factor-β, VDA, vascular damaging agent, CA4P, combretastatin A4 phosphate, HIF, hypoxia inducible factors, MMP, matrix metalloprotease, Tumour, Cancer, Microenvironment, Stroma, Fibroblast, T cell, MicroRNA, Prognostic, Therapeutic

## Abstract

Solid tumours comprise, not only malignant cells but also a variety of stromal cells and extracellular matrix proteins. These components interact via an array of signalling pathways to create an adaptable network that may act to promote or suppress cancer progression. To date, the majority of anti-tumour chemotherapeutic agents have principally sought to target the cancer cell. Consequently, resistance develops because of clonal evolution, as a result of selection pressure during tumour expansion. The concept of activating or inhibiting other cell types within the tumour microenvironment is relatively novel and has the advantage of targeting cells which are genetically stable and less likely to develop resistance. This review outlines key players in the stromal tumour microenvironment and discusses potential targeting strategies that may offer therapeutic benefit.

****Focal points**:**

• Benchside
  ○ The tumour stroma consists of mesenchymal, immune and vascular cells housed in an extracellular matrix. Stromal cells and extracellular matrix proteins represent genetically stable targets which can be exploited in cancer treatment. Numerous *in vitro* and animal studies support the concept of stromal-directed treatment.
• Bedside
  ○ Several therapeutic strategies have been developed or repurposed to target the stroma. The anti-angiogenic agent bevacizumab was one of the first specific stromal-targeting agents to be licensed for cancer treatment over a decade ago. More recently, immune modulation of the stroma has become a hugely successful strategy, with novel drugs such as checkpoint inhibitors set to revolutionise cancer treatment.
• Governments
  ○ Funding bodies should continue to acknowledge the pivotal role that the stroma plays in cancer progression, in parallel with cancer cell itself. Undoubtedly, the most successful treatment regimens of the future will address both the “seed” and the “soil”.

## 1 Introduction

Paget [1] first highlighted the importance of the tumour microenvironment (TME) over a century ago when he described his ‘seed and soil’ hypothesis. The concept that cancer cells (seeds) require a specific TME (soil) in order to establish or propagate a tumour is just as valid today and is indeed recognised as the first key milestone, in a series of articles by the journal Nature, highlighting the most influential discoveries in the field of cancer [2].

**Fig. 1:**
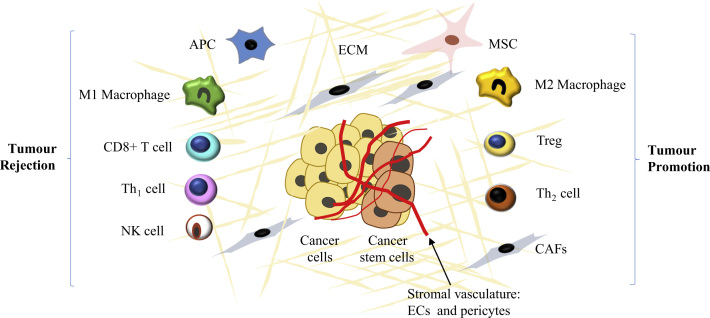
Cellular composition of the tumour microenvironment with relevance to tumour promotion and rejection. APC – antigen presenting cell; ECM – extracellular matrix; MSC – mesenchymal stem cell; Treg – regulatory T cell; Th – helper T cell; CAF – cancer-associated fibroblast; EC – endothelial cell.

The microenvironment of solid tumours consists of a diverse network of cellular and acellular components [3]. A histological categorisation is to divide these elements into cancer and stromal compartments, with the stromal compartment further divided into a cellular component and the extracellular matrix. Cancer cells and cancer stem cells [4] form the cancer compartment. Stromal cells can be sub-classified into: mesenchymal (fibroblasts and mesenchymal stem cells (MSCs)), immune (T cells, macrophages, natural killer (NK) cells and antigen presenting cells (APCs)) and vascular (endothelial cells (ECs) and pericytes). Of these, vascular cells are permanently located in the TME, immune cells are transient, and mesenchymal cells may be permanent or transient [5]. The extracellular matrix (ECM) is a biologically active three-dimensional scaffold for cancer and stromal cells, comprising proteoglycans and fibrous molecules [6]. By its cellular interactions it permits tumour expansion, invasion and dissemination [7]. Fig. 1 summarises key components of the TME.

Malignant cells accrue mutations which can allow escape from regulatory mechanisms [8]. We can think of these cells as genetically unstable and highly plastic [9]. One of the effects of chemotherapy is to apply selection pressure to these heterogeneous cells, allowing expansion of resistant clones. In contrast, stromal cells are not mutated [10], turnover more slowly [11] and are therefore genetically more stable. These cells are less likely to develop chemotherapeutic drug resistance. The stroma is therefore an appealing target for novel cancer therapies.

Cancer is characterised by a misregulation of genes such as those encoding oncogenic, tumour suppressor and DNA repair proteins [12]. As a result, there are certain key signalling pathways which are commonly altered across many cancer types, underpinning the hallmarks of cancer [13]. Notably, microRNAs (miRs) are master regulators of gene expression and signalling pathways, with an estimated one-third of all genes under miR control [14]. As a consequence, there has been much interest in modulating oncogenic and tumour-suppressing miRs for therapeutic benefit.

In this review, we outline existing and potential targets for novel chemotherapeutic agents in the stroma with an introduction to miR targeting strategies.

## 2 The mesenchymal stroma

Fibroblasts are mesenchymal cells which secrete ECM components [15]. Cancer-associated fibroblasts (CAFs) are variably defined in the literature. It is best to consider them as any fibroblast adjacent to the tumour, rather than by their expression profile or cell of origin [16]. CAFs may originate from resident fibroblasts [17], bone marrow-derived MSCs [18] and epithelial cells (including cancer cells and endothelial cells) through the process of epithelial-mesenchymal transition (EMT; [19],[20],[21]). Spindle shaped myofibroblasts expressing alpha smooth muscle actin (αSMA) and vimentin, with typical ultrastructural appearances [22] are a subpopulation of CAFs which are associated with tumorigenesis and cancer progression [23], [24] but it is important to note that not all CAFs are myofibroblasts. Nonetheless, αSMA positivity is most commonly used to denote the ‘activated’ CAF phenotype [25] and TGF-β is widely accepted as the main cancer cell-secreted factor which activates CAFs [26], [17].

CAFs, like other fibroblasts, regulate the integrity of the ECM through their secretory function. In normal physiology, myofibroblasts are capable of closing a wound. Cancer is considered to be a ‘wound that does not heal’ [27] and in this context, myofibroblastic CAFs are thought to remain persistently activated [28]. Activated CAFs have been shown to alter the morphology of epithelial cells [29] and drive tumorigenesis [30].

CAFs express the enzyme lysyl oxidase (LOX) and lysyl oxidase-like proteins (LOX-L) 1–4 which allow crosslinking of ECM substrates such as collagen with elastin. This stiffens the ECM and stimulates integrin-dependent mechanotransduction pathways which promote invasion [31]. LOX/LOX-L expression correlates with worse prognosis in head and neck, lung, ovarian and breast cancers [32]. LOX inhibitors such as beta-aminopropionitrile (BAPN) have been shown to reduce breast cancer cell motility *in vitro* [33]. In cervical cancer models, the same drug was shown to reduce hypoxia-induced EMT, invasion and migration [34]. Bondareva et al. [35] showed that BAPN reduced metastasis of MDA-231 breast cancer cells only if given at the same time or prior to systemic injection of tumour cells. This suggests that LOX inhibition is important in preventing extravasation of tumour cells from the circulation. Nonetheless, this class of drug has not been carried forward into human trials as yet. Inevitably, with collagen crosslinking being a widespread and physiological process, the side effect profile of this kind of drug may be difficult to accept. The key will be to identify isotypes of the enzyme which are specific to CAFs and the TME.

CAFs also play an important role in angiogenesis by secreting fibroblast growth factor (FGF)-2 [36] as well as vascular endothelial growth factor (VEGF; [37]). The selective dual inhibitor brivanib targets FGF and VEGF receptors, which is important because VEGF receptor inhibition alone with bevacizumab has encountered chemoresistance [38]. Huynh and colleagues [39] showed that brivanib reduced tumour size in human xenografts of hepatocellular carcinoma by increasing apoptosis, and reducing proliferation and microvessel formation. Phase 2 trials showed efficacy as first [40] and second line (after sorafenib; [41]) treatment in advanced hepatocellular carcinoma. In a randomised phase 3 trial in patients with unresectable hepatocellular carcinoma, brivanib improved time to extrahepatic/vascular spread and time to radiological progression but not overall survival when used as an adjuvant to trans-arterial chemoembolization [42].

Bone marrow cells are important in determining CAF transdifferentiation and stromal histology. Systemic endocrine signals such as osteopontin from tumours recruit particular bone marrow cells (Sca1^+^cKit^-^) into the circulation. The activated bone marrow cells secrete granulins creating a desmoplastic myofibroblast-containing stroma around indolent responding tumour cells, stimulating their expansion [43]. Theoretically, inhibiting the activation of Sca1^+^cKit^−^ cells and or granulin secretion seems appealing. Unfortunately, this has not materialised into any human studies which directly target CAFs. As Bateman [44] points out, Elkabets’ work is contrary to our understanding that removal of the primary tumour can stimulate development of metastases from dormant responding cells. Furthermore, selective inhibition of granulins presents a problem because their cell surface markers are poorly defined [45].

Another therapeutic strategy is to limit the accumulation of CAFs in the TME. The anti-fibrotic agent pirfenidone has been shown to reduce the proliferation of primary pancreatic stellate cells *in vitro*. Oral administration of this agent to mice with subcutaneous and orthotopic tumours containing pancreatic cancer and stellate cells reduced tumour growth and metastasis respectively [46]. This drug, originally purposed for idiopathic pulmonary fibrosis, is not yet in human trials for cancer but there have been phase 2 trials in the treatment of neuromas in neurofibromatosis type 1 [47].

Another important cell type in the mesenchymal stroma is the MSC. These are pluripotent circulating cells which are recruited to the TME [48]. A significant proportion of CAFs are derived from MSCs [49]. Soluble factors secreted by cancer cells are thought to promote this transdifferentiation [50]. Karnoub and colleagues [51] showed that when MSCs are co-injected with weakly metastatic breast cancer cells in a human xenograft model, it significantly increases their metastatic potential. Breast cancer cells are thought to provoke CCL5 secretion by MSCs, which increases their own motility, invasion and metastatic potential. Furthermore, there is evidence to suggest that MSC-derived CCL5 promotes EMT in a variety of breast cancer cell lines [52]. A similar effect on APS gastric cancer cells has been observed [53]. The CCR5 receptor antagonist maraviroc (Celsentri) was shown to reduce the total body burden of primary and secondary prostate tumours in mice [54]. Interestingly there is no record of any human study of maraviroc in prostate cancer on the US Clinical Trials database. However, there has been a single centre phase 1 trial for maraviroc in colorectal liver metastasis, the results of which are pending (NCT01736813).

## 3 The immune stroma

Typical immune cells in the TME include T cells, macrophages, NK cells and APCs. These cells have opposing effects on cancer progression either through immune surveillance or by allowing immune tolerance [55]. M1 macrophages, CD8+ cytotoxic T cells, T helper 1 (Th1) cells, NK cells and APCs have predominantly anti-tumour effects whereas M2 macrophages, regulatory T cells (Tregs) and Th2 cells may support tumour progression [56], as represented in Fig. 1.

CD8+ T cells bind to ‘non-self’ antigens presented by host MHC class 1 molecules through the T cell receptor, triggering apoptosis in host cells, including cancer cells [57]. High levels of CD8+ tumour-infiltrating lymphocytes (TILs) have been shown to predict better outcomes in various cancers such as melanoma [58], ovarian [59], colorectal [60], breast [61] and head and neck [62].

Adoptive T cell therapy has been widely studied in the context of metastatic melanoma. In one of the initial human studies, a cohort of patients with refractory disease underwent chemotherapeutic lymphodepletion followed by autologous transfer of rapidly expanded CD8+ T cells. 18 of 35 patients showed objective clinical and/or radiological responses (3 complete responses) with a mean duration of 1 year. There were no treatment related deaths but the expected haematological toxicities were observed. However, the only patient who was EBV seronegative at recruitment became seropositive and developed a lymphoma 4 months after treatment. Importantly, persistence of CD8+ cells after transfusion determined the degree of efficacy in this study [63]. Genetic modification of T cells to improve tumour reactivity using T cell receptor [64] and chimeric antigen receptor [65] now offers a potential cure to patients with metastatic melanoma. The challenge now is to translate these approaches to other cancer types. Adoptive T cell therapy has been reviewed extensively elsewhere [66], [67]. We now focus on other T cell targets.

Tregs are CD4+/CD25+ T cells specifically expressing FoxP3 which are capable of suppressing the effects of other immune cells [68], [69]. Several studies have shown that high numbers of intratumoral Tregs are associated with advanced stage or recurrence in various malignancies including ovarian [70], breast [71], oesophagogastric [72] and liver [73]. Specific targeting of Tregs has shown promise in animal and early human studies. Mice bearing a renal cell carcinoma were given anti-CD25 monoclonal antibody against Tregs and inoculated with pre-primed CD8+T cells. At day 50, tumours were undetectable in all the mice. In contrast, untreated mice, or mice treated with either CD8+ T cells or anti-CD25 did not survive [74]. Rech et al. [75] repurposed the anti-CD25 monoclonal antibody daclizumab (FDA-approved for prophylaxis of organ rejection) to the same effect. As well as downregulating Tregs, daclizumab was shown to reprogramme them to express the pro-inflammatory cytokine interferon-γ (IFN-γ). In a small phase 1 study of metastatic breast cancer patients, daclizumab reduced Treg numbers within 1 week, a phenomenon which lasted for at least 7 weeks. Samples from all evaluable patients showed greater CD8+T cell response to at least one tumour antigen (hTERT peptide) after daclizumab treatment and vaccination. However, when comparing cohorts who received daclizumab plus vaccination or vaccination alone, although immune response rate and overall survival was greater in the combination cohort, this was not statistically significant.

The PI3K–AKT pathway is an important regulator of Treg activity. Selective inhibition of the PI3Kδ isoform has been shown to repress AKT activation and proliferation of Tregs *in vitro* and *in vivo* in a TC-1 tumour model [76]. More comprehensively, Ali et al. [77] knocked down PI3Kδ in mice and showed reduction in primary tumour growth in melanoma, lung, thymoma and breast xenografts and reduction of metastasis when 4T-1 breast cancer cells were injected systemically. Knockdown mice had reduced numbers of Tregs in draining lymph nodes when injected with 4T-1 cells and allogenic Treg transfer from one knockdown animal to another upregulated intratumoral CD8+ T cells in the thymoma model. Furthermore, pharmacological inhibition of PI3Kδ with PI-3065 had similar effects to knockdown in the breast cancer model and improved survival in a pancreatic cancer model. Interestingly, idelalisib, the PI3Kδ inhibitor has been approved for the treatment of B cell malignancies [78], [79], [80] but not necessarily with the intention of modulating Tregs.

Immune checkpoints are important regulators of CD8+ and Treg cell activity. In the cancer setting, a vast number of new antigens are generated. To prevent autoimmunity, immune checkpoints are activated to dampen pro-inflammatory T cell responses, at the cost of allowing immune escape of cancer cells [81]. The most well known immune checkpoints are cytotoxic T lymphocyte-associated protein-4 (CTLA-4) and programmed cell death protein-1 (PD-1). CTLA-4 mainly prevents co-stimulation of the CD28 receptor [82] and represses early activation of CD8+ T cells in lymphoid tissues. PD-1 attenuates CD8+ T cell activity in peripheral tissues [83] such as the TME. Both CTLA-4 and PD-1 promote Treg development and activity [84]. The introduction of immune checkpoint inhibitors in the treatment of metastatic melanoma has been greeted with a great deal of enthusiasm. An important phase 3 trial of advanced melanoma patients showed that the CTLA-4 inhibitor ipilimumab significantly improved overall survival with or without co-administration of the melanoma antigen gp100 [85]. In a more recent randomised controlled trial, the PD-1 inhibitor nivolumab, as monotherapy or in combination with ipilimumab, effectively improved progression-free survival compared to ipilimumab monotherapy. As expected in patients with PD-1 ligand negative tumours, combination therapy was more effective than monotherapy [86]. Similarly, targeting the PD-1 ligand (PD-L1) with the monoclonal antibody atezolizumab has proved efficacious with tolerable side effects in a phase 1 trial of metastatic melanoma, non-small cell lung cancer (NSCLC) and renal cell cancer, in patients with tumours expressing high total and high CD8+ T cell levels of PD-L1 [87]. Similar results were seen in a phase 1 trial of metastatic urothelial bladder cancer [88]. This drug is now in phase 2 studies for metastatic NSCLC (e.g. POPLAR trial [89]). Additionally, mismatch repair deficiency is associated with a high somatic mutational burden. This generates greater numbers of neoantigens, necessitating enhanced checkpoint inhibition. Le et al. [90] conducted a phase 2 trial of the PD-1 inhibitor pembrolizumab (FDA-approved for melanoma and NSCLC [91],[92]) in patients with mismatch repair deficient and proficient metastatic cancers, with a focus on colorectal cancer. Patients with mismatch repair deficient tumours responded better to PD-1 inhibition, providing proof of principle for this concept.

Th cell subtypes have antagonistic effects on cancer progression. Th1 cells activate cytotoxic CD8+ T cells and have been shown to mediate tumour regression in a murine pulmonary fibrosarcoma model [93]. In murine B cell lymphoma and myeloma models of successful immunosurveillance, Th1-associated cytokines (IL2, IL12 and IFN-γ) were consistently identified in implanted tumour-Matrigel plugs [94]. In contrast, Th2 cells are involved in immune tolerance and have been shown to be markers of active [95] and advanced [96] disease. There may be a role for Th2 cells in prognostication.

In terms of macrophages, classical M1 polarisation (IL-12^high^IL-10^low^) is associated with expression of pro-inflammatory cytokines and tumour rejection, whereas alternative M2 polarisation (IL-12^low^IL-10^high^) is associated with tumour progression [97], [98]. Tumour-associated macrophages (TAMs) are a mixed population of M1 and M2 cells, although some [99] suggest that they generally possess the M2 phenotype because they are incapable of activating sufficient nitric oxide and pro-inflammatory cytokine responses to tumour cells [100]. Furthermore, TAMS are associated with Treg activation [70], PD-L1-mediated checkpoint activation [101], angiogenesis [102] and invasion [103]. Many studies have shown that stromal TAMs predict poor prognosis in cancers such as lung [104], endometrial [105], thyroid [106] and breast [107]. Indeed, a recent meta-analysis of stromal TAMs in cancer prognosis showed worse overall survival in gastric, urogenital and head and neck cancers but surprisingly, a better overall survival in colorectal cancer [108].

In order to selectively target TAMs, Luo et al. [109] produced a DNA vaccine against legumain, a stress protein which TAMs overexpress. In the prophylactic group, pulmonary metastases from intravenously injected breast, colon and NSCLC cells were significantly reduced following vaccination. In the therapeutic group, overall survival was significantly better if animals were vaccinated after orthotopic injection of breast cancer cells. The survival of TAMs is dependent on colony stimulating factor receptor-1 (CSFR-1). Ries and colleagues [110] blocked CSFR-1 dimerisation with a novel monoclonal antibody *InRG7155 (emactuzumab). vitro*, this resulted in cell death of TAMs. *In vivo*, this led to reduction of TAMs with an associated increase in CD8+ T cells and was associated with less tumour growth in animal models of colorectal cancer and fibrosarcoma. In a small phase 1 study, administration of this agent effected at least a partial metabolic response in all 7 patients with diffuse-type giant cell tumour. More recently, Cassier et al. [111] reported an objective response in 24 of 28 patients with grade 3 adverse events in 5 of 25 patients.

NK cells are innate to the immune system and are able to directly kill tumour cells in several different *in vitro* cancer models [5]. NK cells express the death ligands FasL and TRAIL which bind to Fas and DR5 receptors on target cells to trigger apoptosis [112]. Alternatively, NK cells express CD16 which mediates antibody dependent cell-mediated cytotoxicity [113], [114]. However, MHC class 1 molecules on tumour cells are able to bind killer inhibitory receptors on NK cells to dampen their cytotoxic effects [115]. This suggests that cytotoxic T cell activation is at the expense of NK activity. Nonetheless, NK cell immunotherapy has been popular in several recent human studies, the majority of which concern haematological malignancies (summarised in [116]). Different methods of enhancing NK activity include *in vivo* cytokine stimulation and adoptive transfer of *ex vivo*-stimulated autologous, allogenic or NK cell lines. Early phase 2 studies used IL-2 to stimulate resident NK cells and proved to be effective in metastatic melanoma [117] and metastatic renal cell carcinoma [118]. However, this was associated with severe side effects including sepsis-related mortality. In terms of adoptive therapy, Ishikawa et al. [119] conducted a small phase 1 study with malignant gliomas. Peripheral blood mononuclear cells (PBMCs) were isolated from each patient and NK cells were expanded *ex vivo* using IL-2. Autologous NK cells were then injected into the tumour cavity and/or intravenously in a total of 16 courses. MRI showed partial responses after 3 of the 16 courses. Importantly, there were no significant neurological side effects. Another phase 1 study in patients with NSCLC used allogenic NK cells from donor relatives, expanded *ex vivo* with IL-15 and hydrocortisone. There was partial response in 2 of 16 patients and disease stabilisation in 6 patients. Again, there were no major local or systemic side effects [120]. Infusion of the cell line NK-92 has also proved to be well tolerated in patients with a range of advanced malignancies with a persistence of at least 48 h [121]. The requirement to generate large numbers of NK cells for immunotherapy has now driven research into NK cell production from embryonic stem cells [122].

APCs process foreign antigens and present them alongside MHC class 1 or 2 molecules to naïve CD8+ and Th cells respectively. Professional APCs such as dendritic cells, macrophages and B cells are so named because they process and present antigens most effectively [123]. In terms of cancer therapy, APCs have been used to improve the efficacy of adoptive T cell transfer. The ideal adoptive treatment will use T cells which proliferate, persist, target and destroy tumour cells [124]. Autologous and artificial APCs have been used to this effect, however the use of autologous APCs is cumbersome and time consuming [125]. Artificial APCs have been generated using Drosophila cells [126], murine fibroblasts [127] and K562 human leukaemic cells [128]. The overarching principle is to produce a cell which expresses restricted HLA antigens in combination with transfected co-stimulatory molecules such as ICAM-1 (CD54) and B7.1 (CD80). Alternatively, magnetic beads embedded with HLA antigens [129] and HLA expressing extracellular vesicles [130] have been employed instead of feeder cells in the experimental setting. More recently, Butler and colleagues [131] transfected K562 cells with HLA-A2, CD80 and CD83 to produce aAPC-A2 cells, from which they selected a single clone. This clone was used to expand autologous MART-1 specific CD8+ T cells from PBMCs *ex vivo*. The MART-1 T cells were then given to 9 patients with advanced melanoma in a total of 17 infusions. This therapy has the benefit of not requiring lymphodepletion or IL-2 treatment and consequently there were no severe adverse effects. One patient had a complete metabolic response which lasted 54 months and 4 others had stabilisation of disease to at least day 70.

## 4 The vascular stroma

The tumour vasculature consists of ECs and pericytes. ECs form stromal capillaries and pericytes provide structural support. In healthy tissue, pericytes intimately cover ECs and through the expression of VEGF and angiopoetin-1 they lead to increased EC survival and structural stabilisation [132]. Reciprocally, ECs express platelet-derived growth factor-β (PDGF-β) and recruit pericytes from the stroma [133]. In the TME, tissue hypoxia and the consequent upregulation of pro-angiogenic factors such as VEGF and angiopoetins has the effect of loosening the connections between pericytes and ECs [134]. Ultimately, pericytes detach completely and this allows a disordered budding of new capillaries which underlies angiogenesis [135]. PDGF receptor antagonists targeting pericytes have been shown to stunt growth of end stage pancreatic islet cell tumours in mice [136]. However, the beneficial effect on the primary lesion seems to be at a cost. The Kalluri group has shown that inhibiting pericytes in an invasive breast cancer model has two detrimental effects: firstly it reduces pericyte coverage of ECs which correlates directly with metastasis; secondly, it aggravates tissue hypoxia which drives the EMT/mesenchymal–epithelial transition cascade [137]. Given this evidence, it seems prudent not to target pericytes but to focus on their downstream angiogenic signals.

It is well established that hypoxia develops as a tumour expands and that its size is limited without neovascularisation or angiogenesis. Folkman et al. [138] first reported that a soluble factor, now known as VEGF, was responsible for angiogenesis. VEGF is released by pericytes and binds to VEGF receptors on ECs which become the “tip” of a sprouting chain. The “tip” migrates towards the highest VEGF concentration which is present in the most hypoxic regions of the TME. ECs which lie behind the “tip” bind to each other through surface ligand-receptor interactions and form a new capillary [139].

Several monoclonal antibodies have been developed to target VEGF-driven angiogensis. Bevacizumab, targeting VEGF-A, is the most well known amongst these. It received US FDA approval in 2004 for use in metastatic colorectal cancer in combination with standard chemotherapy [140]. Since then it has been used in advanced NSCLC [141], renal, ovarian [144] and cervical [145] cancers, supported by evidence from large phase 3 studies. However, there are a certain group of patients who do not respond to treatment or develop resistance [146]. Fan et al. [147] showed that long term exposure (3 months) of colorectal cancer cell lines to bevacizumab led to increased expression of VEGF-A,B and C, increased phosphorylation of VEGF-1 and 2 receptors, increased invasion and migration and increased metastasis when injected into an *in vivo* model. Moreover, VEGF inhibition has mostly had clinical success in combination with traditional chemotherapy, possibly because it normalises stromal vessels and allows better drug delivery [148]. Nonetheless, anti-VEGF agents are still being developed. For example, the VEGF-2 receptor monoclonal antibody ramucirumab is licensed for use in advanced gastric cancer after phase 3 trials showed survival benefit as a single therapy (REGARD trial [149]) and in combination with paclitaxel (RAINBOW trial [150]).

Another class of anti-angiogenic drugs are the VEGF/PDGF-receptor tyrosine kinase inhibitors which have shown significant response in several phase 3 trials. These drugs inhibit tyrosine kinase receptors from activating intracellular serine/threonine kinases such as Raf, resulting in reduced proliferation and angiogenesis [151]. Sorafenib is an oral multikinase inhibitor which is approved for use as monotherapy in advanced renal cell, hepatocellular and thyroid carcinomas. The first large phase 3 study of sorafenib monotherapy in 2007 showed increased progression free survival compared to placebo in advanced renal cell carcinoma [142], [143]. However, there was significantly more hypertension and angina in the treatment arm. Another randomised controlled trial showed an increase in overall survival and time to radiological progression in advanced hepatocellular carcinoma patients [152]. Most recently, an increase in progression free survival has been shown in radioactive iodine-refractory differentiated thyroid cancer [153].

Whereas anti-angiogenic drugs target new vessel formation, vascular damaging agents (VDAs) target existing vessels, causing ischaemia and haemorrhagic necrosis of the tumour [154]. There are two classes of VDAs: small molecule microtubule targeting drugs and ligand based drugs. Small molecule agents exploit differences between tumour and normal vessels such as greater proliferation and reliance on a cytoskeleton. Ligand based drugs target proteins such as VEGF-receptors which are overexpressed in tumour vessels [155]. Combretastatin A4 phosphate (CA4P) or fosbretabulin, is an example of a small molecule VDA. CA4P binds to tubulin causing microtubule depolymerisation [156]. CA4P has reached phase 2 studies for advanced anaplastic thyroid carcinoma where there was poor efficacy [157] and relapsed ovarian carcinoma where significant clinical responses deemed suitable an extension to the trial [158]. These studies corroborated phase 1 trial safety data suggesting that it is a safe drug overall with common severe side effects of neuropathy and tumour pain.

An interesting ligand based approach is to fuse toxins to stromal vascular ligands. Rosenblum's group constructed the fusion molecule VEGF(121)/rGel which combines a VEGF ligand with the plant toxin gelonin [159]. This has shown promise in reducing tumour growth in animal models of bladder [159], metastatic breast [160] and metastatic prostate cancer [161]. Drugs of this class are yet to make clinical trials.

Reduced oxygen tension in the TME leads to upregulation of hypoxia inducible factors (HIFs) by ECs [162]. HIF-1 regulates EC proliferation [163] and HIF-2 causes EC senescence [164]. Branco-Price and colleagues [165] showed that there was slower migration of tumour cells through HIF-1α deficient EC layers and reduced metastasis in HIF-1α deficient mice. HIF-2α deletion has the opposite effects. Consequently, digoxin has been found to inhibit HIF-1α ([166]). It is currently in a phase 2 study which aims to assess tissue HIF1α levels in resected breast cancers after 2 weeks of digoxin pre-treatment (NCT01763931).

## 5 ECM

The ECM is comprised of proteoglycans such as hyaluronan and versican and fibrous proteins such as collagen, elastin, fibronectin, laminin, periostin and tenascin-C [167], [168]. It is biologically active and plays a role in cellular adhesion, migration, proliferation and survival [169]. ECM composition varies between tissues. In the cancer setting, fibroblasts express vast amounts of ECM proteins leading to tissue stiffening [7]. Stiffening is exacerbated by LOX-mediated collagen crosslinking as described above [31]. Paszek and colleagues [170] suggest that matrix stiffness is an exogenous force whilst Rho-dependent cytoskeletal tension is an endogenous force on cancer cells. The summation of these forces results in clustering of integrins and activation of ERK and ROCK signalling which leads to increased proliferation and contractility respectively. The pro-inflammatory reaction in the TME triggers myofibroblast transdifferentiation which adds to fibrosis or desmoplasia [171]. Desmoplasia has been associated with poor prognosis in cancer for over 20 years [172]. Additionally, matrix metalloproteases (MMPs) are expressed by stromal and epithelial cells and remodel the ECM, particularly the basement membrane and potentiate release of growth factors such as VEGF [173]. Clearly then, the combined ECM effects of stiffness, reciprocal contractility, desmoplasia and MMP activity are important in tumorigenesis and cancer progression. Below we outline some key ECM targets.

Hyaluronan is associated with a permissive TME [174]. The dense hyaluronan matrix surrounding cancer cells makes it difficult for chemotherapeutic drugs to penetrate. This is a particular problem for monoclonal antibody therapy because it prevents antibody directed cell-mediated cytotoxicity by NK cells. Singha et al. [175] showed that co-administration of recombinant hyaluronidase with the monoclonal antibody traztuzumab and NK cells significantly reduced tumour growth in ovarian cancer xenografts. Recombinant hyaluronidase (PEGPH20) has been successfully profiled for safety in phase 1 trials in advanced pancreatic cancer [176]. There is currently an ongoing phase 2 trial of PEGPH20 in untreated stage 4 pancreatic carcinoma in combination with paclitaxel and gemcitabine (NCT01839487).

The matricellular protein periostin is a ligand for αvβ3 and αvβ5 integrins on epithelial cells, promoting cell motility [177]. Underwood et al. [178] showed that periostin is associated with poor overall survival and disease-free survival in oesophageal adenocarcinoma. Additionally, periostin was shown to be secreted by CAFs and had the effect of activating the AKT survival pathway in oesophageal cancer cells. Periostin is also upregulated in colorectal primary and secondary tumours [179]. *In vitro*, periostin was shown to directly increase proliferation of several colorectal cancer cell lines. This effect was attenuated by addition of a periostin-specific antibody which triggered cancer cell apoptosis and worked synergistically with 5FU. Animal studies have shown that MZ-1, a monoclonal antibody to periostin, can reduce growth and metastatic potential of A2780 ovarian cancer xenografts [180]. Currently there are no ongoing clinical studies of anti-periostin therapy.

Decorin was shown to be differentially expressed in the tumour mass of malignant angiosarcomas compared to benign haemangiomas [181]. Grant et al. [182] transfected sarcoma and carcinoma cell lines with decorin. These cells produced significantly less VEGF than their wild type counterparts. Conditioned media from the transfected cells reduced EC attachment, migration and differentiation. *In vivo*, decorin transfected xenografts were smaller and showed less neovascularisation. Recently, Xu et al. [183] created an oncolytic adenovirus carrying the decorin gene (Ad.dcn) which significantly reduced bony metastases in a murine prostate cancer model. Decorin manipulation has not matured into a useable therapy as yet.

Tenascin-C is preferentially expressed by various tumours [184]. Monoclonal antibody therapy with 81C6 has reached phase 2 trials in patients with malignant gliomas showing favourable efficacy when compared to brachytherapy or radiosurgery [185]. In this study, 33 patients had injection of radioiodine-labelled 81C6 to cerebral resection cavities followed by standard chemoradiotherapy. Median survival was better in this cohort than in historical controls receiving standard treatment. However, 9 patients developed haematological toxicity and 5 patients had neurological toxicity. Consequently, this drug has not featured in phase 3 studies. An alternative to antibody therapy is the use of RNA interference (RNAi) to downregulate tenascin-C. In one study, double stranded RNA targeting tenascin-C (ATN-RNA) was injected into the resection cavities of 46 patients with malignant brain neoplasms. This treatment showed survival benefit in astrocytomas and glioblastomas [186].

## 6 MiR signalling in the TME

MiRs are small (20–30 nucleotide) non-coding RNAs which repress protein translation by binding to the 3’ UTR region of mRNAs [187]. Several physiological processes such as proliferation, differentiation and apoptosis are regulated by miRs [188]. Deregulated miRs can lead to tumorigenesis [189]. There has been a great deal of interest in deregulated miRs as biomarkers in diagnosis, tumour subtyping, prognosis and response to treatment. MiR-21 overexpression is associated with breast, hepatocellular and colorectal carcinoma for example [190]. MiR-21 represses tumour suppressor proteins such as PDCD4 and PTEN [191]. We have shown by laser capture microdissection that miR-21 is a stromal rather than epithelial signal in colorectal tumours. Indeed, co-injection of miR-21 overexpressing fibroblasts with DLD-1 colorectal cancer cells leads to increased metastasis in an orthotopic murine model [192]. To explain this phenomenon, it is possible that miR-21 is shuttled from fibroblasts to cancer cells. This is supported by evidence that RNAs can be transferred between cells in extracellular vesicles [193].

There are several observational studies assessing miR profiles in different malignancies. One systematic review of over 40 studies highlighted total (stromal and epithelial) tumoral miR-21 overexpression and let-7 downregulation as key determinants of patient outcome [194]. Indeed, we have shown that stromal miR-21 predicts poor disease free survival and overall survival in stage II (node negative, metastasis negative) colorectal cancer [195]. This is important because it may help to distinguish those stage II patients who require adjuvant chemotherapy from those who do not.

In terms of miR-based cancer therapy, the miR-34a mimic MRX34 is currently in a phase 1 trial for patients with unresectable, advanced or metastatic hepatocellular carcinoma which is due to complete in 2016 (NCT01829971). MiR-34 is a tumour suppressor which is thought to oppose proliferation, migration and chemoresistance [196]. MRX34 uses a liposomal delivery system which allows the drug to accumulate in the liver [197]. There are no other miR based cancer therapies in human trials at present, however the miR-122 antagonist Miravirsen for hepatitis C is in late phase 2 trials [198]. Similarly, the anti-miR-21 agent RG-012 (Regulus Therapeutics) has received orphan drug status from the US FDA and European Commission and is in a phase 1 trial for the treatment of renal fibrosis in Alport's syndrome.

Table 1 summarises the key experimental and clinical studies which have led to the development and approval of stromal-directed therapies in solid tumours.

**Table 1:** Stromal targeting therapies in solid tumours: a summary of relevant clinical and pre-clinical studies. CAF – cancer-associated fibroblast; LOX – lysyl oxidase; BAPN – beta-aminopropionitrile; FGF – fibroblast growth factor; VEGF – vascular endothelial growth factor; MSC – mesenchymal stem cell; TCR – T cell receptor; CAR – chimeric antigen receptor; PD-1 – programmed cell death protein-1; CTLA4 – cytotoxic T lymphocyte associated protein-4; NSCLC – non-small cell lung cancer; PD-L1 – PD-1 ligand; Treg – regulatory T cell; mAb – monoclonal antibody; TAM – tumour-associated macrophage; CSFR1 – colony stimulating factor-1; APC – antigen presenting cell; EC – endothelial cell; GOJ – gastro-oesophageal junction; PDGF – platelet-derived growth factor; VDA – vascular disrupting agent; HIF – hypoxia inducible factor; ECM – extracellular matrix; miR – microRNA.

***Stromal compartment***	***Target cell/molecule***	***Drug/therapy***	***Mechanism of action***	***Level of evidence***	***Cancer type***	***US FDA approval***	***Key reference(s)***
Mesenchymal	CAF	BAPN	LOX inhibitor	Pre-clinical	Breast		[31,35]
		Brivanib	FGF/VEGF receptor antagonist	Phase 3	Hepatocellular		[42]
		Pirfenidone	Antifibrotic	Pre-clinical	Pancreatic		[46]
	MSC	Maraviroc	CCR5 antagonist	Phase 1	Colorectal		NCT01736813 (clinicaltrials.gov)
Immune	CD8+ T cell	Autologous T cells	Adoptive T cell	Phase 2	Melanoma		[63]
		TCR T cells	Adoptive T cell	Phase 1/2	Melanoma		[64]
		CAR T cells	Adoptive T cell	Pre-clinical	Melanoma		[65]
		Nivolumab	PD-1 inhibitor	Phase 3	Melanoma	Yes	[86]
		Pembrolizumab	PD-1 inhibitor	Phase 2/3	Mismatch repair deficient tumours; Melanoma; NSCLC	Yes (melanoma; NSCLC)	[90,^91^,92]
		Ipilimumab	CTLA-4 inhibitor	Phase 3	Melanoma	Yes	[85]
		Atezolizumab	PD-L1 inhibitor	Phase 2	NSCLC		[89]
	Treg	Daclizumab	CD25 mAb	Phase 1	Breast		[75]
		PI-3065	PI3Kδ inhibitor	Pre-clinical	Breast; Pancreatic		[77]
	TAM	Emactuzumab	CSFR1 antagonist	Phase 1	Diffuse type giant cell tumour		[111]
	NK Cell	IL-2	Resident NK stimulation	Phase 2	Melanoma; renal		[117,118]
		Autologous NK cells	Adoptive NK cell	Phase 1	Glioma		[119]
		Allogenic NK-92 cells	Adoptive NK cell	Phase 1	Various		[121]
	APC	Artificial aAPC-A2 cells	MART-1 T cell generation	Phase 1	Melanoma		[131]
Vascular	EC/Pericyte	Bevacizumab	VEGF receptor antagonist	Phase 3	Colorectal; NSCLC; renal; ovarian; cervical	Yes (all)	[140,^141^,^144^,145]
		Ramucirumab	VEGF receptor antagonist	Phase 3	Gastric; GOJ	Yes (gastric; GOJ)	[149,150]
		Sorafenib	VEGF/PDGF receptor inhibitor	Phase 3	Renal; hepatocellular; thyroid	Yes (all)	[142,^152^,153]
		Fosbretabulin	Small molecule VDA	Phase 2	Thyroid; Ovarian		[157,158]
		VEGF(121)/rGel	Ligand-based VDA	Pre-clinical	Bladder; Breast; Prostate		[159,^160^,161]
		Digoxin	HIF-1 alpha inhibitor	Phase 2	Breast		NCT01763931 (clinicaltrials.gov)
	Pericyte	SU6668	PDGF receptor antagonist	Pre-clinical	Pancreatic		[136]
ECM	Hyaluronan	PEGPH20	Recombinant hyaluronidase	Phase 1b/2	Pancreatic		[176]; NCT01839487 (clincaltrials.gov)
	Periostin	MZ-1	Periostin mAb	Pre-clinical	Ovarian		[180]
	Decorin	Ad.dcn	Oncolytic virus	Pre-clinical	Prostate		[183]
	Tenascin-C	81C6	Tenascin-C mAb	Phase 2	Glioma		[185]
		ATN-RNA	RNA interference	Phase 1	Glioma		[186]
MiR	MiR-34	MRX34	MiR-34 mimic	Phase 1	Hepatocellular		NCT01829971 (clinicaltrials.gov)

## 7 Conclusion

For a long time, Paget's theories about the “soil” remained in the shade and ignored. In recent years however, there has been a focus of research efforts in this field, simultaneously bringing to light a variety of stromal-directed therapeutic strategies. The great appeal of the stromal TME is its genetic stability and reduced likelihood of Darwinian emergence of resistance, as seen in cancer cells. Moreover, stromal-directed therapy offers two key benefits: firstly, it creates an arid “soil” making it more difficult for a tumour to establish at both primary and secondary sites; consequently, it reduces the required doses of traditional cytotoxic chemotherapeutic drugs. With this in mind, the future of cancer therapy looks promising. The ultimate treatment regimens will include interventions which manipulate each component of the TME, in both stromal and cancer compartments, to promote tumour rejection. Ideally, these regimens will be personalised for each individual depending on the cellular and molecular fingerprint of their TME. This is not unrealistic if we consider that adoptive immune cell therapy, which epitomises personalised cancer treatment, is already well established.

In this review, we have highlighted key cellular and molecular targets within the stromal TME and summarised relevant preclinical and clinical data associated with each. The article provides up to date, structured and comprehensible information on the translational aspects of stromal cancer therapy and how it has evolved from bench to bedside.

## Executive summary

• The TME is a functional ecosystem of cancer cells and stroma which interact through an array of signalling molecules. The stroma consists of mesenchymal, immune and vascular cells together with the ECM.
• In comparison to cancer cells the stroma is genetically stable and therefore, anti-stromal therapies are less likely to succumb to chemoresistance.
• There is huge excitement about redirecting and modulating the immune stroma to reject a tumour with the most successful approaches being adoptive T cell therapy and immune checkpoint inhibition respectively.
• Targeting the vascular stroma with anti-VEGF agents is associated with intrinsic resistance. This has accelerated FDA approval of alternative anti-angiogenic agents such as sorafenib and prompted the development of mechanistically different drugs (VDAs).
• The mesenchymal stroma and ECM are equally important in cancer progression but therapies specifically targeting CAFs, MSCs and ECM proteins are fewer and in comparatively earlier stages of development.
• MiRs are master controllers of gene expression which are deregulated in cancer. MiR profiling has been shown to be useful in cancer prognostication. MiR modulating therapies are now in early human trials.

## Ethical statement

This is a review article and does not refer to any unpublished human or animal studies which we have undertaken.

## Contributors

RB and AM conceptualised the article. RB, AS and AM designed the structure of the article. RB, MB, AS and AM drafted the article with RG, HA, JP and GT revising critically for intellectual content. All authors have approved the final article.

## Funding source

RB is funded by a Cancer Research UK (CRUK) Clinical Research Training Fellowship and a CRUK pre-doctoral bursary (A20526). CRUK made no direct contribution to study design; collection, analysis and interpretation of data; in the writing of the manuscript; or in the decision to submit the article for publication.

## Conflicts of interest

None declared.
